# Self‐organized criticality in human epidemiology

**DOI:** 10.1063/1.2008613

**Published:** 2005-07-20

**Authors:** Nico Stollenwerk

**Affiliations:** Mathematics Department, Porto University, Portugal NIC, Research Center Jülich, Germany; School of Biological Sciences, Royal Holloway University London, UK; University of Granada; University of Granada; University of Granada

## Abstract

As opposed to most sociological fields, data are available in good quality for human epidemiology, describing the interaction between individuals being susceptible to or infected by a disease. Mathematically, the modelling of such systems is done on the level of stochastic master equations, giving likelihood functions for real live data. We show in a case study of meningococcal disease, that the observed large fluctuations of outbreaks of disease among the human population can be explained by the **theory of accidental pathogens**, leading the system towards a critical state, characterized by power laws in outbreak distributions. In order to make the extremely difficult parameter estimation close to a critical state with absorbing boundary possible, we investigate new algorithms for simulation of the disease dynamics on the basis of **winner takes all** strategies, and combine them with previously developed parameter estimation schemes.

## THEORY OF ACCIDENTAL PATHOGENS

Many micro-organisms live in their hosts mostly unnoticed and only sometimes cause problems to the hosts but more important to their own disadvantage. The hosts' problems in form of a disease are thus an accident of the normally unharmful micro-organisms, hence behaving as comensals under normal circumstances an only rarely become pathogens accidentally.

This scenario of accidental pathogens is best studied in cases where the disease is grave and the comensals omni-present, as in meningococcal disease. The accidental pathogen is found in large proportions of the host population, harmless carriage meningococci being as high as 20 to 30 percent in humans in certain age classes even in industrialized societies. Only rarely disease is caused, but then the symptoms are severe and often life threatening in meningitis and septicaemia caused by meningococci. The accepted clinical picture is that the bacteria live in the nasopharynx of their hosts without any noticable symptoms and are normally cleared off by the host's immune system after a short period of time. In order to escape the immune system the bacteria are highly mutable, especially in their membrane proteins, which are also responsible for attaching the bacteria to the nasal wall towards the blood stream. Here the mechanisms are highly disputed and subject to intensive research in medicine and microbiology (private communication, Martin Maiden). Clear again is the stage that when the meningococci have survived long enough in the host's blood stream or the meninges to release their metabolic by-products the host will suffer life threatening poisoning. The period between harmful acquisition of the bacteria (infection from other hosts) and the poisoning lasts only around a day.

CP779, *Modeling Cooperative Behavior in the Social Sciences: Eighth Granada Lectures*
edited by P. L. Garrido, J. Marro, and M. A. Muñoz
© 2005 American Institute of Physics 0-7354-0266-3/05/$22.50

**FIGURE 1.** Rosenbluth method (straight lines) versus direct simulations (dashed lines) for a birth death process after 30 discrete time steps. The Rosenbluth method gives already at first inspection a much more regular curve than the direct method.

A model capturing especially the feature of mutation between harmless and potentially pathogenic infection shows that large outbreaks of disease (with power law distribution) occur when the pathogenicity (probability to cause a disease case) is small (Stollenwerk, Jansen (2003) [2]). The fact that a smaller probability to cause disease can lead to larger outbreaks is counter-intuitive when not taking the harmless carriage also into account, leading to near critical fluctuations.

It has also been demonstrated that in a situation when the harmless bacteria mutate to any pathogenicity between zero and a reasonable maximal value, the distribution of pathogenicities develops towards smaller and smaller values (Stollenwerk, Jansen (2003) [3]), hence the system evolves towards criticality. Subsequently, a first inspection of outbreak data of meningococcal disease in England and Wales has shown hints of such a scenario (Stollenwerk, Maiden, Jansen (2004) [4]).

To a further test the hypothesis of accidental pathogens on real data of outbreaks not only the parameter pathogenicity but also the duration of carriage (and eventually other parameters) have to be estimated reliably. Because of the closeness to criticality, the outbreaks are highly variable, and hence difficult to estimate with standard procedures. In the literature, duration of carriage has been estimated between 11 months and a few weeks. The months estimates derive from carriage studies in schools, whereas outbreak data show seasonality and even short time adjustment to hosts' interactions (Stollenwerk, Maiden, Jansen (2004) [4]).

## PARAMETER ESTIMATION NEAR ABSOBING STATES

Previously, a method for parameter estimation for epidemiologic models, using stochastic simulations in the case there is no analytic solution available, has been suggested (Stollenwerk, Briggs (2000) [6]), but it runs into troubles here due to too many simulations being absorbed while scanning parameters near criticality. On the other hand, recent progress in obtaining rare events close to absorbing boundaries by artificially preventing absorbtion and subsequently reweighting of the realization, applied successfully to protein folding problems (e.g. Grassberger, Nadler, 2000, [7]), provides a promissing

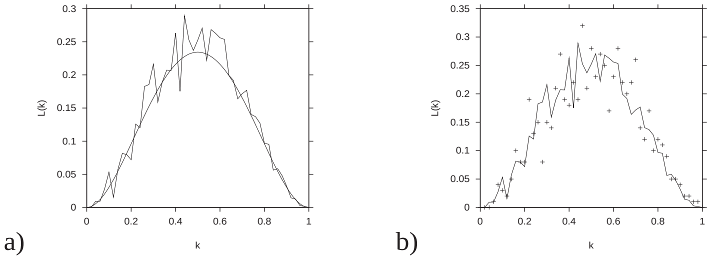

**FIGURE 2.** Likelihood function for parameter estimation in the birth and death process described in the text. Simulated likelihood with Rosenbluth step versus analytic likelihood, and simulated likelihood with Rosenbluth step versus likelihood from direct simulations. The direct simulations (crosses) tend to deviate more from the in this case known analytic solution.

way to speed up the parameter estimation also in situations such as described above in epidemiology.

The basic models for accidental pathogens are based on master equation formulation of stochastic epidemiologic systems and in some analysis reducible to simple birth-death processes (Stollenwerk, Jansen, 2003, [2]), but with several state variables and parameters (SIRYX-model), whereas for testing purposes the parameter estimation method (called "$\eta$-ball method, Stollenwerk, Briggs, 2000, [6]) was initially applied to a simpler two state variable model (SI-model), but later also successfully used in a wider context of an SEIRX-model (Stollenwerk, 2001, [8]).

To test the performance of the approach investigated here, we apply it to a simple cellular automaton of a birth-death process, where properties of the system near criticality are well known in many aspects (Grassberger, de la Torre, 1979, [9]), and first apply the $\eta$-ball method plainly to long unabsorbed runs, then include the so called Rosenbluth step (Rosenbluth and Rosenbluth, 1955, [10]) in the simulations of the parameter estimation part. This mimics the fact that outbreaks of large sizes in epidemiology are easily noticed by the recording personnel, whereas fast absorbed realisations often go unnoticed or are misinterpreted.

## ACKNOWLEDGMENTS

We thank Walter Nadler, Peter Grassberger, Friedhelm Drepper, all Jülich, Vincent Jansen, London, Martin Maiden, Oxford, and Alberto Pinto, Porto, for instructive discussions on various topics of the present work.
